# A Computational Approach for Identifying Synergistic Drug Combinations

**DOI:** 10.1371/journal.pcbi.1005308

**Published:** 2017-01-13

**Authors:** Kaitlyn M. Gayvert, Omar Aly, James Platt, Marcus W. Bosenberg, David F. Stern, Olivier Elemento

**Affiliations:** 1 Institute for Computational Biomedicine, Department of Physiology and Biophysics, Weill Cornell Medicine, New York, NY, United States of America; 2 Institute for Precision Medicine, Weill Cornell Medicine, New York, NY, United States of America; 3 Tri-Institutional Graduate Program on Computational Biology and Medicine, New York, NY, United States of America; 4 Department of Pathology, Yale University School of Medicine, New Haven, Connecticut, United States of America; 5 Department of Dermatology, Yale University School of Medicine, New Haven, Connecticut, United States of America; Rutgers University, UNITED STATES

## Abstract

A promising alternative to address the problem of acquired drug resistance is to rely on combination therapies. Identification of the right combinations is often accomplished through trial and error, a labor and resource intensive process whose scale quickly escalates as more drugs can be combined. To address this problem, we present a broad computational approach for predicting synergistic combinations using easily obtainable single drug efficacy, no detailed mechanistic understanding of drug function, and limited drug combination testing. When applied to mutant BRAF melanoma, we found that our approach exhibited significant predictive power. Additionally, we validated previously untested synergy predictions involving anticancer molecules. As additional large combinatorial screens become available, this methodology could prove to be impactful for identification of drug synergy in context of other types of cancers.

## Author Summary

While targeted therapies have achieved remarkable antitumor responses, resistance to targeted agents frequently develops and renders the targeted drug ineffective. Combination therapies have been successful in delaying resistance and overcoming resistance. Additional goals of combination therapy are to achieve enhanced effectiveness through drug synergy and to reduce the frequency and severity of adverse events through lower individual drug dosage levels. However the identification of synergistic drug combinations is often a labor and resource intensive process. Therefore a systematic method for identifying optimal combinations would be highly impactful. Here we present a computational method for predicting synergistic and effective drug combinations using only single drug efficacy information. We have applied and validated our method on a high-throughput drug screen of 780 combinations involving 40 individual molecules in the context of mutant BRAF melanoma. Additionally we have made predictions and validated 11 previously untested drug combinations with a diverse set of outcomes.

## Introduction

Targeted therapies designed to specifically target molecules involved in carcinogenesis have achieved remarkable antitumor efficacy. In melanoma, over half of patients are reported to harbor activating mutations in the BRAF oncogene [1, 2]. BRAF inhibitors, such as vemurafenib, have been developed to selectively kill mutant BRAF positive cells [3]. Patients initially exhibited significant responses to these drugs, with 48% responding to vemurafenib in phase 1 and 2 clinical trials, however resistance developed within months [3].

Combination therapy has been proposed for preventing and overcoming resistance. This is thought to be a promising option because resistance to the combinatorial therapy would require either acquisition of multiple mutations rapidly [4] or an individual mutation that is able to bypass both drugs [5], both of which are low probability events. Additional goals of combination therapy are to lower drug dosage levels in order to reduce the frequency and severity of adverse events and to achieve enhanced effectiveness through either drug additivity or synergy [4].

Drug synergy can occur through a variety of mechanisms. These include enhancement of bioavailability, through inhibition of parallel pathways[6], and chemosensitization, in which the first compound primes the cells to be sensitive to the second drug[7]. Synergy is generally quantified through either effect based or dose-effect based methodologies. Effect-based methods compare the independent effects of drugs, while dose-effect based methods assume nonlinear individual dose–effect curves[8]. The most popular effect based method is the Bliss Independence model, which assumes that drugs act independently and the expected additive effect is based on the common probabilistic independence formula[8]. However limitations of this approach include that it does not account for nonlinearity in dose response curves [9] and its independence assumption. Since the mechanism of action for many drugs remain unknown, the validity of the independence assumption is often not met [8]. A popular dose-effect based method is the Chou-Talalay Combination Index (CI), which is a median-effect equation based on the “mass-action law”[10]. A major limitation of the Chou-Talalay method is its dependence on accurate and well-defined dose-effect curves, which are not always available[8].

Given the poor prognosis of BRAF melanoma and the rapid rate at which resistance develops and tumors progress, there is an urgent need to identify suitable combinations. The first drug combination for treatment of advanced melanoma was approved in January 2014 and involved the BRAF inhibitor, dabrafenib, and the MEK inhibitor trametinib. This combination was pursued due to the great response rates of the individual drugs with the goal of preventing drug resistance. Indeed, it has been observed that the combination delays the development of resistance and prolongs progression free and overall survival[11]. However it is certain that many more combinations exist and are not yet known. Additionally, a subset of patients that were treated with the combination of dabrafenib and trametinib have developed resistance to this combination therapy[11].

Existing methods that have been developed to predict synergistic combinations have generally relied on mechanistic insights. However they have been applied only in limited specific contexts, such as in B cells [7]. Furthermore numerous studies have shown that synergy is very dependent on context [2, 6, 7]. This makes it difficult to utilize any prior knowledge about synergistic drug combinations from other different cancers or genotypes. A systematic method for identifying optimal combinations would therefore be highly impactful. Here we propose a computational approach utilizing existing high-throughput drug screen data to help identify other combinations that are both synergistic and effective in the context of mutant BRAF melanomas.

## Results

### Single Dose Response Predictive of Combinatorial Synergy and Effectiveness

We set out to determine whether combination efficacy and synergy could be predicted from single agent efficacies. Previous computational approaches to drug combinations have shown that the dose response curves of single agents exhibit predictive power for identifying synergistic combinations [7]. To further investigate this, we utilized a high-throughput drug screen that was performed by Held *et al*. [2]. In this study, the response of 150 single agents and a large combinatorial drug screen involving 40 drugs were experimentally tested across mutant BRAF, mutant RAS, and wild-type BRAF and RAS (WT) cell lines. For each drug pair, we derived a feature set that consisted of the mean and difference of the single agent dose response in each tested cell line. The single agent dose response was represented as the percent of concentration required to inhibit 50% of growth inhibition (GI_50_). Altogether, this resulted in a total of 54 total features representing the similarity of a drug pair’s efficacies in 27 melanoma cell lines (**S1 Fig**).

The results of the combinatorial drug screen were used to identify genotype-selective and synergistic combinations. Genotype-selective combinations were defined to be those that yielded an average 15% or greater growth inhibition exclusively in the genotypic group and achieved at least 50% growth inhibition within the genotypic group. We further defined a general effective combination to be one that achieved at least 70% growth inhibition. Finally we computed synergy labels for each combination using the Chou-Talalay synergy combination index (CI) metric. We decided to use the Chou-Talalay approach because the Chou-Talalay CI was provided in the original dataset and furthermore the mechanism for many drugs in the training set is unknown. We defined a synergistic combination to be a pair of drugs that demonstrated CI < -1 at any concentration level. Overall, the combinatorial screen helped identify 248 BRAF-selective and 161 synergistic combinations.

**Fig 1:**
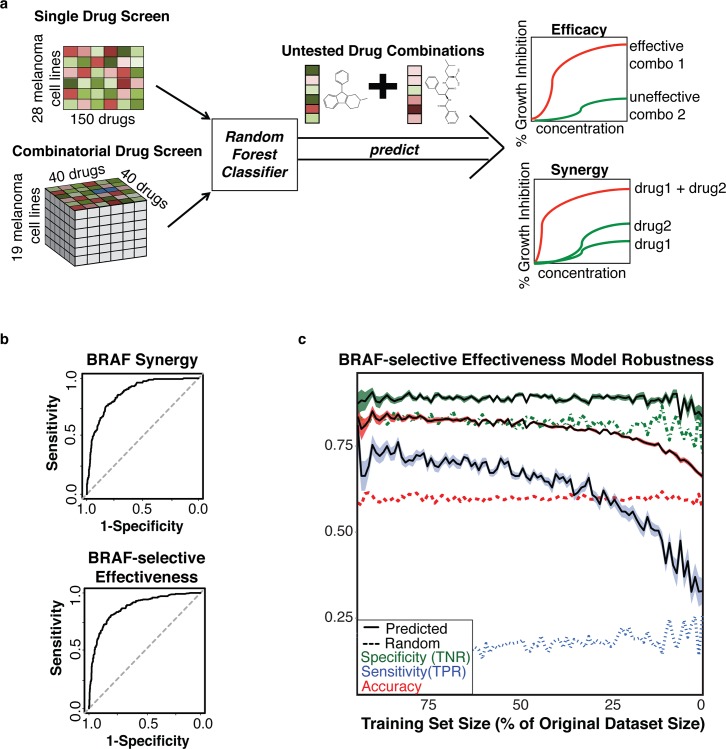
Method schematic and Evaluation of individual model performance. A Our approach integrates a large single drug screen of 150 drugs with a combinatorial drug screen, which tested 780 combinations of 40 unique drugs. This was used to train a random forest model that predicts synergy and genotype-selective efficacy for untested drug combinations. B Receiver operating characteristic (ROC) curves for 10-fold cross-validation of the BRAF-specific effectiveness (top) and synergy (bottom) models. C The effect of randomly removing samples on model accuracy, sensitivity and specificity. At 25% of the original number of combinations was used to train the model, the approach maintained 77.56% accuracy, 89.27% specificity, and 54.91% sensitivity.

We then trained random forest models [12] on 780 drug combinations for each of the outcomes described above in context of BRAF and RAS melanomas (**Fig 1A, S2 Fig**). We evaluated our approach using 10-fold cross-validation and found that our model exhibits significant power (**Table 1**) for predicting both synergy (AUC = 0.8663, Accuracy = 0.8213) and genotype-selective efficacy (AUC = 0.8809, Accuracy = 0.8230) in context of BRAF melanomas (**Fig 1B**). Importantly both models maintained high specificity rates (0.9494 and 0.8894 for synergy and effectiveness respectively) which suggests that there would be few false leads. As a control to identify limitations of our approach, we evaluated our model by making predictions for “sham combinations”, which were cases in which a drug is combined with itself. We found that 98% (147/150) of the sham combinations were predicted to not be synergistic. We also found that both the effectiveness and synergy models exhibit significant robustness (**Fig 1C**). At 25% of the original number of combinations used in the training set, the BRAF-specific effectiveness approach maintained 77.56% accuracy, 89.27% specificity, and 54.91% sensitivity. This suggests that fewer combination testing could be performed while maintaining strong confidence in the positive predictions, given that the specificity remains high. Since high-throughput screens require significant resources and time, this type of approach could prove to be valuable in screening the larger space of drug combinatorial pairs given that a suitable representative set is chosen for initial testing.

**Table 1:** Model Performance

	AUC	Accuracy	Sensitivity	Specificity
**BRAF-specific effectiveness**	0.8809	0.8230	0.6911	0.8894
**General BRAF-effectiveness**	0.8630	0.7800	0.6818	0.8418
**BRAF synergy**	0.8683	0.8213	0.4196	0.9494

The single agent screens performed by Held *et al* [2] included 110 drugs that were not tested in the combinatorial screen, so we applied our approach to the 10,395 additional untested combinations. We predicted 842 combinations to be synergistic, 890 to be effective, and 304 to be both effective and synergistic in context of mutant BRAF melanoma. We found that our predictions had noticeable patterns of synergy and effectiveness (**S3A Fig**). Predicted synergistic combinations involved drugs that had varying levels of efficacy across the different mutant BRAF cell lines, with synergistic combinations demonstrating a trend towards lower correlation of GI50 values across the mutant BRAF cell lines (*p* = 0.07929, Kolmogorov-Smirnov Test). In contrast, combinations involving drugs with similar efficacy profiles across the different cell lines were generally predicted to be non-synergistic. Thus it appears that our approach drew its strength from the large number of tested cell lines.

To further evaluate our approach, we compared these predictions to an independent high-throughput screen that tested 5,778 combinations involving 108 drugs at two concentration levels, high and low[13]. Our prediction dataset contained 274 combinations that overlapped with this independent dataset. We found that our predicted effective combinations had a significantly higher growth inhibition levels than our predicted non-effective combinations (*p =* 0.002602, Student’s t Test) (**S3B Fig**).

### Experimental Validation of Novel Synergistic and Effective Combinations for BRAF Melanoma

**Fig 2:**
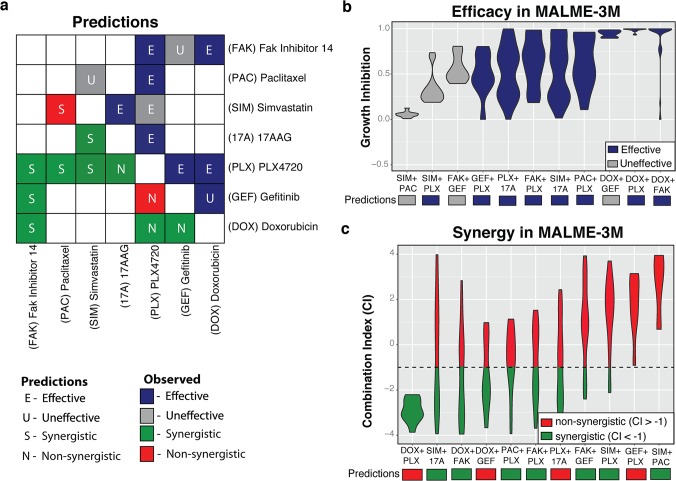
Experimental Validation of Predicted BRAF Effective and Synergistic Combinations. A We selected a set of 11 combinations involving 7 drugs with a diverse set of predictions for experimental validation. These included traditional chemotherapeutic agents (doxorubicin, paclitaxel), targeted agents (PLX4720, gefitinib, FAK Inhibitor 14), a statin (simvastatin), and an antitumor antibiotic (17AAG). B Each drug was tested in combination at medium, and high concentrations, estimated from their GI_10_, GI_25_, and GI_50_ values respectively. The observed growth inhibition levels for all dosage level combinations involving each tested drug combination are shown in violin plots. Violin plots that are colored navy blue are those whose third quantile values where 70% or greater. The predictions for each combination are shown below the plot, with dark blue representing an effective prediction and grey representing an ineffective prediction. C For each tested drug combination, the Chou-Talalay synergy scores were calculated. The observed synergy scores for all dosage level combinations involving each tested drug combination are shown in violin plots. The predictions for each combination are shown below the plot, with green representing a synergy prediction and red representing a non-synergy prediction.

We next identified a subset of 7 drugs with a diverse set of predictions (**S3C Fig**). These drugs included a BRAF inhibitor (PLX4720), a statin (Simvastatin), two chemotherapies (Doxorubicin, Paclitaxel), and three drugs of other various mechanisms (Fak Inhibitor 14, Gefitinib, 17AAG). We tested each of these drugs both alone and in combination in the mutant BRAF melanoma cell line MALME-3M at low, medium, and high concentrations, estimated from their GI_10_, GI_25_, and GI_50_ values respectively. We found that our method continued to demonstrate significant predictive power when tested on cell lines that were independent of the original training set (**Fig 2A**, **Table 2**). We validated 82% and 64% of the effectiveness (**Fig 2B**) and synergy predictions (**Fig 2C**) respectively. Importantly, we also found that the false discovery rates (FDR) for both synergy and effectiveness predictions remained relatively low (14.3% for synergy, 12.5% for effectiveness) despite being tested in a different setting.

**Table 2:** Experimental Validation

	Accuracy	Sensitivity (TPR)	Specificity (TNR)	FDR
**BRAF effectiveness**	0.73	0.71	0.75	0.125
**BRAF synergy**	0.64	0.67	0.5	0.14

**Fig 3:**
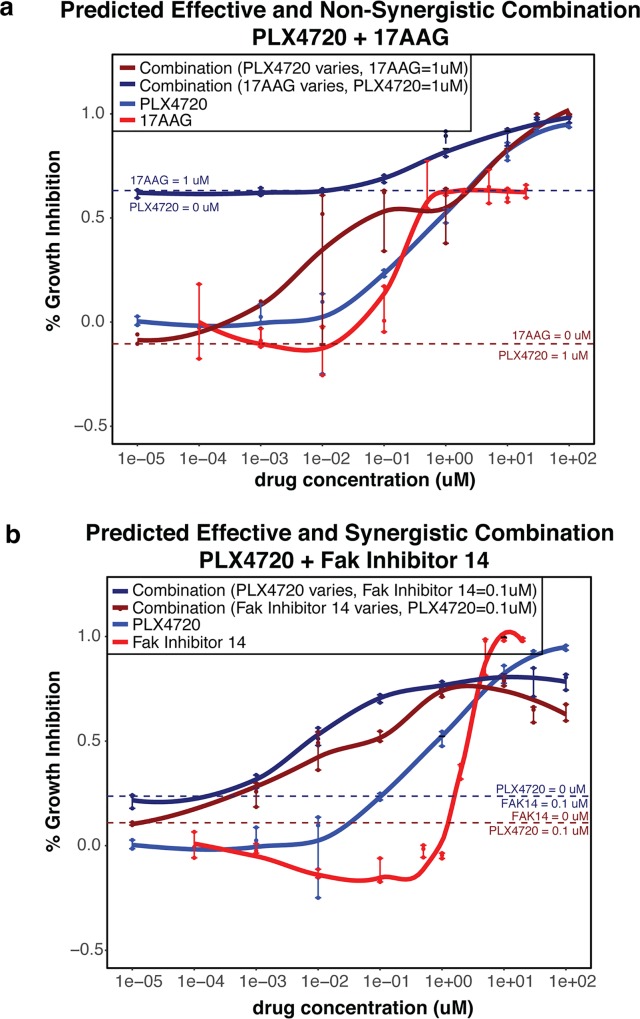
Identification of Synergistic Combinations involving the BRAF Inhibitor PLX4720. A The observed growth inhibition levels for PLX4720 alone (blue) 17AAG alone (red), PLX4720 varying while 17AAG held constant at 1uM (navy blue), and 17AAG varying while PLX4720 held constant at 1 uM (dark red). B The observed growth inhibition levels for PLX4720 alone (blue) FAK Inhibitor 14 alone (red), PLX4720 varying while FAK Inhibitor 14 held constant at 0.1uM (navy blue), and FAK Inhibitor 14 varying while PLX4720 held constant at 0.1 uM (dark red).

BRAF inhibitors are of high interest for treating BRAF-mutant melanomas due to their selectivity and effectiveness. To further investigate the efficacy and synergy of combinations involving PLX4720, we performed more extensive experiments for predicted synergistic and non-synergistic drug partners. PLX4720 was predicted to be synergistic with FAK inhibitor 14 and non-synergistic with 17AAG, a Hsp90 inhibitor. Instead we found both combinations to be synergistic when tested across the 9 combinations of varying concentrations. Interestingly, we observed that while 17AAG appeared to be synergistic when combined with PLX4720 held at a constant rate, PLX4720 itself was not synergistic when 17AAG was held constant (**Fig 3A**). However PLX4720 was very synergistic when combined with a constant dosage of FAK Inhibitor 14 (**Fig 3B**), consistent with the synergy prediction. Additionally we observed that PLX4720 was highly synergistic with paclitaxel. This combination represents a potentially impactful combination since paclitaxel and vemurafenib are both used in clinical trials for the treatment of melanoma [3, 14]. Furthermore previous reports have suggested that the combination of BRAF inhibitors with paclitaxel represents a promising therapeutic approach for overcoming resistance in BRAF melanomas [15].

## Discussion

We found that drug synergy and combinatorial effectiveness can be predicted from a relatively small subset of combinations based only upon single drug efficacies. We experimentally validated novel predictions involving 7 drugs in a BRAF mutant cell line with FDR<0.15. This analysis included compounds that span a variety of drug classes, including targeted therapies and chemotherapies. Additionally, an analysis of the model robustness suggested that it is possible to confidently make these predictions with even smaller subsets, while maintaining significant confidence in the positive predictions. We note that while we propose that a smaller training set can be used to infer these combinations, it is critical to retain a distinctive and representative set of drug combinations in the training set.

The classification errors, particularly those involving synergy predictions, may be due in part to varying genetic conditions since the combinations were tested in a different cell line than the original training set. This is supported by one combination that was tested both in our experiments and in the larger combinatorial drug screen that had inconsistent synergy and effectiveness levels. The combination of Fak Inhibitor 14 and gefitinib was found to be synergistic in the combinatorial screen, however we found it to be non-synergistic in our experiments. Consequently we believe that our false discovery rate would have been even lower if we had tested the combinations in the same setting that was used to generate the training set. However the validation of the majority of our predictions across slightly variable contexts is highly relevant for the treatment of patient cancers, in which the treated patient populations involve different individuals and thus have slightly different genomic profiles than the population under which the therapy was conceived. Additionally we would like to emphasize that any effectiveness (or synergy) predictions that our model makes are in the context of mutant BRAF cell lines. While we hope that some of these findings may be translatable to human patients, many other factors must first be considered.

It is important to note that we did not consider maximum tolerated doses (MTD) in our analysis. A retrospective analysis revealed that there were two drugs included in our analysis that were tested at levels above MTD for humans: Obatoclax and Tamoxifen. Additionally there are many drugs included in the study do not have known MTD. This is because these drugs are experimental and thus have not yet had this evaluated in clinical trials[16]. While we do not believe that this biases the model, it does highlight the importance of clear model interpretation. In particular, we observed in our experiments that synergy generally did not occur at high dosage levels (**Fig 3**), which further suggests that the model would not be biased by including drugs that were tested above MTD. Importantly our approach allows subsequent experimental studies to be prioritized on promising combinations, which can be focused on more clinically relevant information such as MTD.

The use of synergistic drug combinations has the potential to help prevent and overcome drug resistance. It is hypothesized that the application of drug combinations with initial treatment lower the odds of resistance occurring because it requires multiple mutations to bypass both drugs, which is a lower probability event than each drug individually[4, 5]. This could be particularly impactful for combinations involving BRAF inhibitors, which individually have demonstrated remarkable responses in patients but suffer from the rapid development of resistance[3]. To further explore how models could be used to identify drug combinations that overcome drug resistance, we used our training set to derive a set of 24 effective and 12 non-effective combinations involving vemurafenib in resistant cell lines. We applied the framework of our approach to this small dataset and found that there is an underlying signal for predicting combinations that overcome resistance (AUC = 0.677, Accuracy = 0.697). Thus approaches such as ours may be applicable both to directly predicting combinations that overcome resistance, as well as predicting combinations that may help prevent resistance from developing.

Existing methods have previously found that the inclusion of features representing the biological mechanisms of the drug have been most successful[7]. However this information is often not available. Indeed only 50% (20/40) of the drugs in our training set have information about the drug’s target. Thus our method would likely improve as this type of information becomes more widely available enough to include in the model. However our model was able to exhibit significant predictive power despite this information not being available. We hypothesize that this was due to the large number of cell lines that each single agent was tested in. We found that generally the predicted synergistic combinations involved drugs that had varied single agent efficacies across the different mutant BRAF cell lines.

While we have trained and tested our approach in the context of BRAF mutant melanoma, the approach itself is applicable to other types of cancers. As additional large combinatorial screens become available, this methodology could prove to be impactful for the identification of drug synergy within the larger universe of possible drug combinations.

## Materials and Methods

### The Model

150 Single agent and 780 combinatorial efficacies were obtained from the Held *et al* [2] study. The single agent efficacies were collapsed to their GI_50_ values, which is the concentration of the drug required to inhibit 50% of cell growth. Features representing a drug pair were constructed by taking the mean and difference (**S1 Fig**) of the GI50 values for each of 27 tested cell lines (15 mutant BRAF, 6 mutant RAS, 6 wtBRAF/wtRAS).

The combinatorial results in 19 cell lines (8 mutant BRAF, 6 mutant RAS, and 5 WT) were then used to construct labels for each of the 780 drug pairs. Genotype-selective combinations were defined to be those that yielded an average 15% or greater growth inhibition exclusively in the genotypic group and achieved at least 50% growth inhibition within the genotypic group and a general effective combination was defined to be one that achieved at least 70% growth inhibition. Synergy labels for each combination were determined using the Chou-Talalay synergy combination index (CI) metric. We defined a synergistic combination to be a pair of drugs that yielded a CI less than -1 at any concentration level. A random forest model was then trained on the above-described data and evaluated using 10-fold cross-validation. Predictions were made using the trained model for the 10,395 untested combinations that had single agent efficacy information in the dataset.

### Experimental Validation

#### Melanoma cell culture

Malme-3m, SK-Mel-28, and SK-MEL-2 cell lines were generously donated by the Houvras lab at Weill Cornell Medicine. Cell lines were cultured in basal medium [DMEM (Gibco) supplanted with 10% FBS and 1% penicillin/streptomycin (P/S)] and maintained in a 37° incubator at 5% CO2.

#### Agent screening

Cells were pipetted into 384-well plates at 750 cells per well using a multi-channel pipette (Eppendorf) in 20 ul basal medium, and placed in an 37° incubator at 5% CO2 overnight prior to exposure to the agent. Drug stock plates for single therapy agents were created by serial dilution, generally 1:10 from 10 mmol/L stock. Using a multi-channel pipette, 2.5 ul drug volume from drug stock plates were added to the 384-well cell plates. A total of 0.1% and 10% DMSO was used as negative and positive controls, respectively.

For combinatorial, dual-agent screens, a 2.5 ul volume of Drug X was plated at GI-50 concentration (determined from single-agent screen), and 2.5 ul volume from drug stock plate of Drug Y was added in a range of concentrations. Initial dual agent screening was carried out in 384-well plates at 750 cells per well in 20 ul basal medium Further exploration screening were carried out in 96-well plates, in 100ul basal medium with 3500 cells per well.

All experiments were carried out in triplicates at the Weill Cornell Medicine’s Meyer Cancer Center (WCMC MCC, New York, NY). Cells were exposed to drugs for 72 hours, followed by GI measurement by CellTiter-Glo ATP detection assay (Promega) following the manufacturer’s recommended. A 20 ul volume of basal medium served as a background for luminescence.

#### Prediction evaluation

A combination was considered to be effective if the third quantile value of observed growth inhibition values was at least 70%. Synergistic combinations were those that achieved a Chou-Talalay synergy score of -1 in at least one dosage-level pair.

## Supporting Information

S1 Fig Feature construction schematic. For each drug pair, we combined the drug pair’s efficacies by taking the mean (μ) and difference (Δ) in 27 mutant BRAF (red), mutant RAS (green) and WT (blue) melanoma cell lines.
(TIF)

S2 Fig Feature Importance Analysis. Mean decrease Gini coefficient observed upon feature removal for the top 30 features for the (left) BRAF effectiveness model and (right) BRAF synergy model.
(TIF)

S3 Fig Predictions for Previously Untested Combinations. A We applied our trained model to make predictions for 10,395 additional untested combinations. B Comparison of growth inhibition levels, as reported by Friedman *et al*., for 274 predicted effective or ineffective drug combinations. C We focused on a subset of 7 drugs to experimentally follow-up on our predictions for previously untested combinations.
(TIF)
