# Supplementary figures and images for: A Computational Approach for Identifying Synergistic Drug Combinations
Source: PLoS Comput Biol. 2017 Jan 13;13(1):e1005308. doi: 10.1371/journal.pcbi.1005308 (PMC5234777; doi:10.1371/journal.pcbi.1005308)

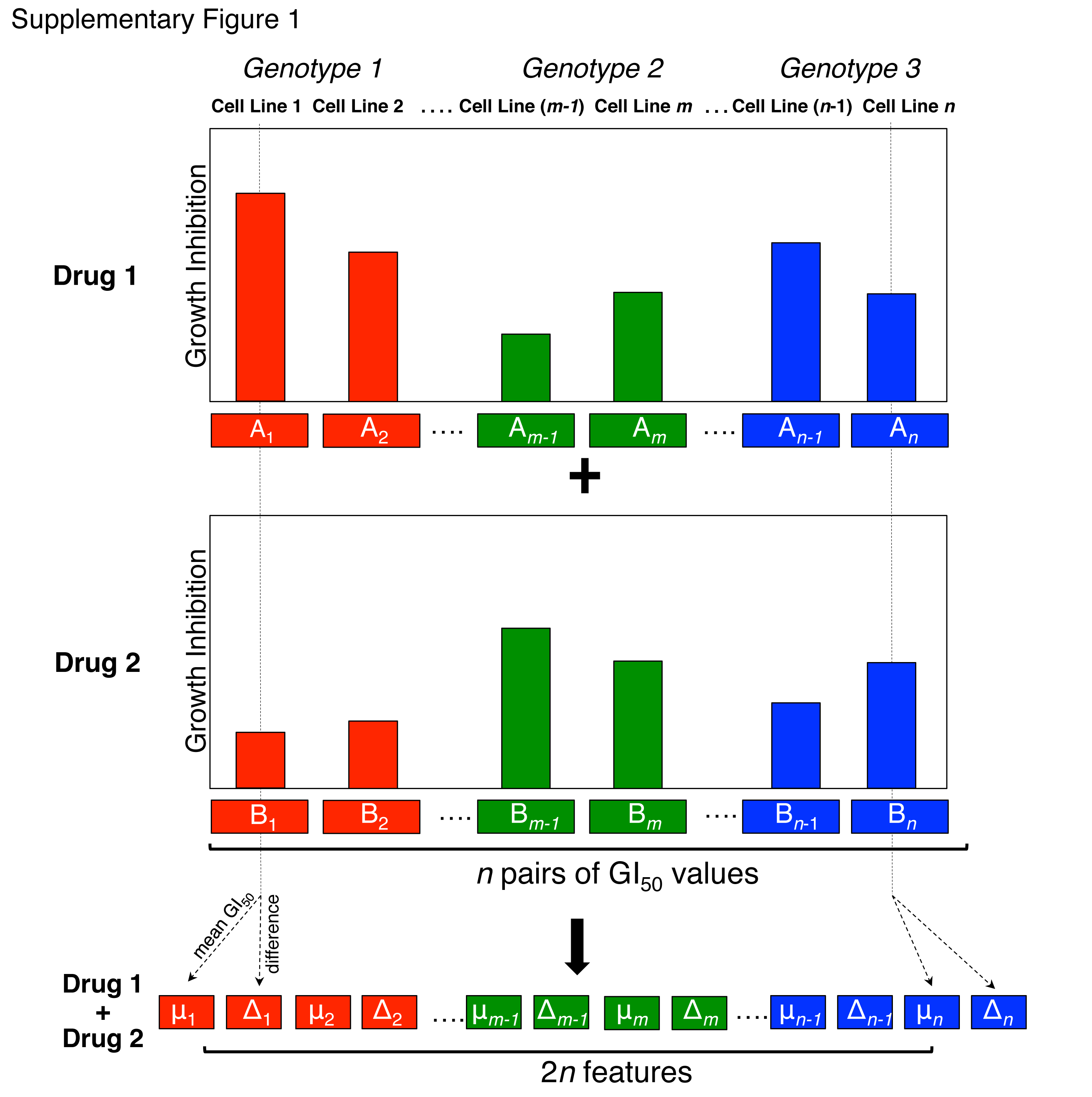

Supplement: S1 Fig — For each drug pair, we combined the drug pair’s efficacies by taking the mean (μ) and difference (Δ) in 27 mutant BRAF (red), mutant RAS (green) and WT (blue) melanoma cell lines. (TIF) [file pcbi.1005308.s001.tif]

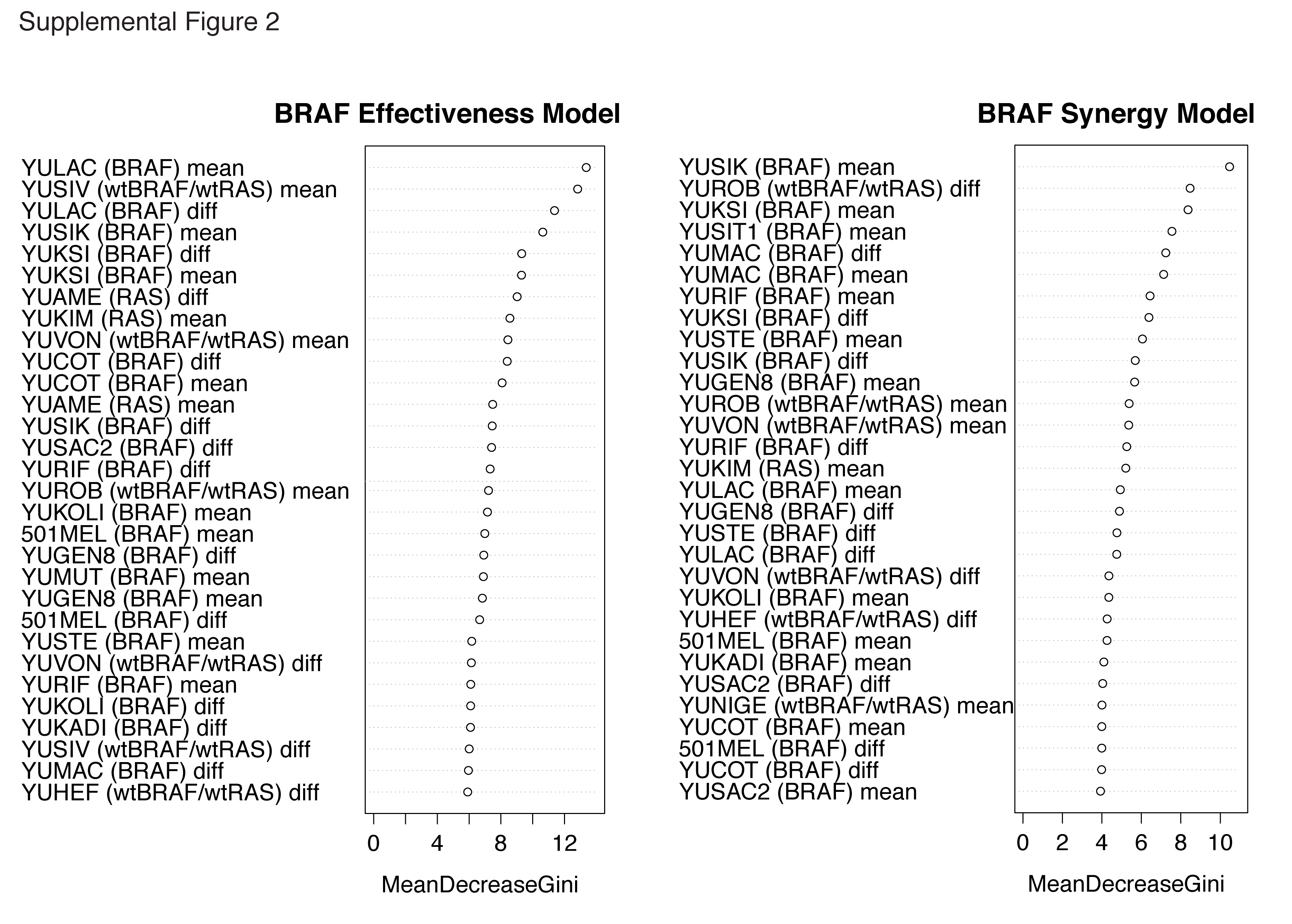

Supplement: S2 Fig — Mean decrease Gini coefficient observed upon feature removal for the top 30 features for the (left) BRAF effectiveness model and (right) BRAF synergy model. (TIF) [file pcbi.1005308.s002.tif]

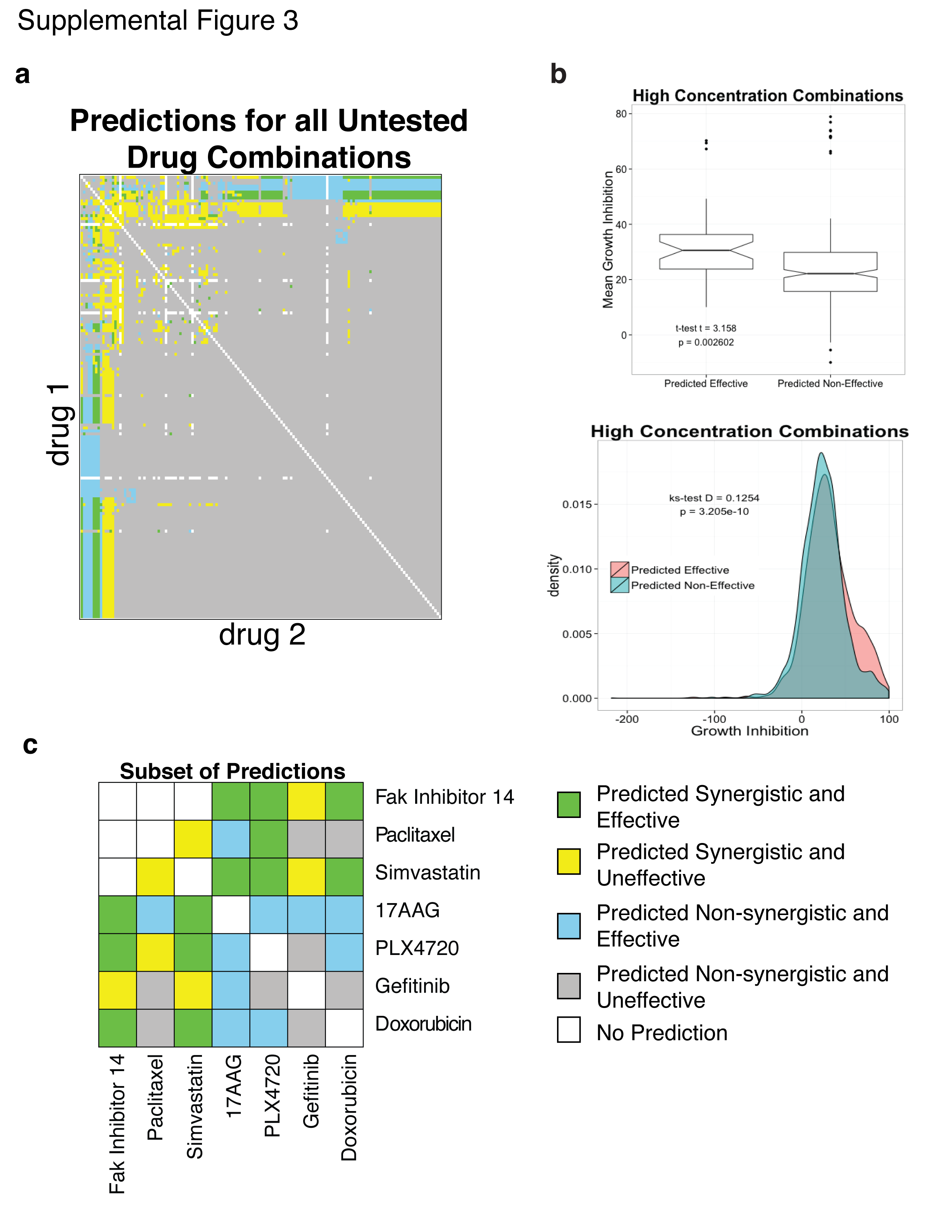

Supplement: S3 Fig — A We applied our trained model to make predictions for 10,395 additional untested combinations. B Comparison of growth inhibition levels, as reported by Friedman et al., for 274 predicted effective or ineffective drug combinations. C We focused on a subset of 7 drugs to experimentally follow-up on our predictions for previously untested combinations. (TIF) [file pcbi.1005308.s003.tif]
